# Bacterial Profile, Antibiotic Sensitivity and Resistance of Lower Respiratory Tract Infections in Upper Egypt

**DOI:** 10.4084/MJHID.2013.056

**Published:** 2013-09-02

**Authors:** Gamal Agmy, Sherif Mohamed, Yaser Gad, Esam Farghally, Hamdy Mohammedin, Hebba Rashed

**Affiliations:** 1 Department of Chest Diseases, Faculty of Medicine, Assiut University, Egypt; 2 Department of Chest Diseases, Faculty of Medicine, El-Minia University, Egypt; 3 Department of Chest Diseases, Faculty of Medicine, Sohag University, Egypt; 4 Department of Clinical Pathology, Faculty of Medicine, Assiut University, Egypt

## Abstract

**Background:**

Lower respiratory tract infections (LRTI) account for a considerable proportion of morbidity and antibiotic use. We aimed to identify the causative bacteria, antibiotic sensitivity and resistance of hospitalized adult patients due to LRTI in Upper Egypt.

**Methods:**

A multicentre prospective study was performed at 3 University Hospitals for 3 years. Samples included sputum or bronchoalveolar lavage (BAL) for staining and culture, and serum for serology. Samples were cultured on 3 bacteriological media (Nutrient, Chocolate, MacConkey's agars). Colonies were identified via MicroScan WalkAway-96. Pneumoslide IgM kit was used for detection of atypical pathogens via indirect immunofluorescent assay.

**Results:**

The predominant isolates in 360 patients with CAP were *S. pneumoniae* (36%), *C. pneumoniae* (18%), and *M. pneumoniae* (12%). A higher sensitivity was recorded for moxifloxacin, levofloxacin, macrolides, and cefepime. A higher of resistance was recorded for doxycycline, cephalosporins, and β-lactam-β-lactamase inhibitors. The predominant isolates in 318 patients with HAP were, *methicillin-resistant Staphylococcus aureus*; MRSA (23%), *K. pneumoniae* (14%), and polymicrobial in 12%. A higher sensitivity was recorded for vancomycin, ciprofloxacin, and moxifloxacin. Very high resistance was recorded for β-lactam-β-lactamase inhibitors and cephalosporins. The predominant organisms in 376 patients with acute exacerbation of chronic obstructive pulmonary diseases (AECOPD) were *H. influnzae* (30%), *S. pneumoniae* (25%), and *M. catarrhalis* (18%). A higher sensitivity was recorded for moxifloxacin, macrolides and cefepime. A higher rate of resistance was recorded for aminoglycosides and cephalosporins.

**Conclusions:**

The most predominant bacteria for CAP in Upper Egypt are *S. pneumoniae* and atypical organisms, while that for HAP are MRSA and Gram negative bacteria. For acute exacerbation of COPD, *H. influnzae* was the commonest organism. Respiratory quinolones, macrolides, and cefepime are the most efficient antibiotics in treatment of LRTI in our locality.

## Introduction

Acute respiratory tract infections, such as bacterial pneumonia and acute exacerbations of chronic bronchitis, account for a considerable proportion of morbidity and antibiotic use. Moreover, these infections result in high mortality rates.^1^ Unfortunately, the three major bacterial respiratory pathogens; *Streptococcus pneumoniae*, *Moraxella catarrhalis* and *Haemophilus influenzae*; have a worldwide increasing prevalence of antibiotic resistance.^2^,^3^,^4^ The importance of monitoring the progress of such resistance has led to numerous international, regional and national surveillance programmes. However, results from surveillance studies show wide variations in susceptibility rates, both geographically and over time.^1^,^5^ Prevalent flora and antimicrobial resistance pattern may vary from region to region depending upon the antibiotic pressure in that locality.^6^ Thus, there is a great need for local resistance prevalence data in order to guide empirical prescription and to identify areas in which medical need for new agents is greater. Therefore, the present study was designed to identify the bacterial profile of lower respiratory tract infections (LRTIs) in Upper Egypt and to determine the antibiotic susceptibility and resistance patterns among these pathogens in our locality.

## Materials and Methods

### Patients

The present multicentre prospective study was performed at three University Hospitals in Upper Egypt (Assiut, El-Minia and Sohag University Hospitals) for 3 consecutive years; from July 2009 to June 2012; aiming to identify the causative bacteria, antibiotic sensitivity and antibiotic resistance in hospitalized adult patients ( > 15 years old) due to lower respiratory tract infections in Upper Egypt. The study included 360 patients with community-acquired pneumonia (CAP), 318 patient with hospital-acquired pneumonia (HAP) and 376 patients with acute exacerbation of chronic obstructive pulmonary disease (AECOPD). The later patients had mild or moderate exacerbations.^3^ All patients were subjected to full clinical, radiological and relevant laboratory examinations. All patients were non-ICU in-patients. CAP was defined as pneumonia acquired outside the hospital setting.^7^ HAP was defined as pneumonia that occurs 48 hours or more after admission, which was not incubating at the time of admission.^8^ AECOPD were defined according to the GOLD guidelines.^3^ Indications to hospitalize patients with CAP or AECOPD were according to the relevant international guidelines for each category,^3^,^4^ respectively. Samples collected from all patients were sent to the Department of Clinical Pathology, Faculty of Medicine, Assiut University. The study was approved by the local ethical committee of the Faculty of Medicine, Assiut University and a written informed consent was obtained from all patients for publication of this study.

### Sample collection

Samples included sputum and/or bronchoalveolar lavage (BAL), for Gram stain and culture, blood samples for blood cultures and serum sample for serology. One morning spontaneously produced or induced sputum sample was obtained from the majority of patients. The valid sputum originating from the lower respiratory tract was defined as that containing squamous epithelial cell less than 10/high power field and polymorphonucleocytes more than 25/high power field. One BAL was taken from each patient under anesthesia. BAL was obtained to diagnose if there was other hidden pathology. We would not normally recommend this as a routine investigation for culture in LRTIs. For blood samples; 5 to 10 ml of venous blood was collected from each patient using sterile syringes. Blood samples were inoculated immediately under complete aseptic conditions into bottles containing 50 ml of brain heart infusion broth.^9^

### Processing of samples

Such validated sputum as well as BAL samples were cultured on three bacteriological media (Nutrient, Chocolate and MacConkey's) agar plates. Sputa or BAL were mechanically liquefied and homogenized by vortexing for 1 minute with glass beads followed by centrifuging at 3.000 revolutions per minute for 10 minutes. Specimens were then serially diluted in sterile 0.9 % saline solutions with final concentrations of 10^−4^, 10^−5^, 10^−6^ and the colonies were counted as number of colonies per plate X 100(dilution factor). The valid sputum culture defined as that had quantitative culture ≥10^5^CFU/ml and the valid BAL culture that had ≥10^4^ CFU/ml.^9^ Plates were incubated aerobically at 37°C with 5% CO2 for 24–48 hours. For blood samples; the blood culture bottles were incubated aerobically at 37°C for 7 days. The bottles were examined daily for evidence of bacterial growth as haemolysis, gas production or turbidity above the red cell line. Subcultures using sterile syringes were done on blood agar, chocolate agar, MacConkey's agar and Bile Esculin Azide agar daily for 7 days before reporting blood cultures as negative.^9^ Isolation of anaerobes was not considered.

### Identification of bacterial isolates

Bacterial isolates were identified by their biochemical characteristics via the automated Microscan WalkAway system (Siemens-France).^10^ Isolates from repeat culture of previously recruited patients and isolates identified as commensals or contaminants were excluded.

### Identification of staphylococcal isolates

According to Louie *et al.*,^11^ staphylococci were identified by standard methods including the gram stain, catalase test and tube coagulase test. Samples were cultured on Mannitol Salt Agar (Oxoid, UK), where S. aureus produces yellow colonies (1 mm in diameter) surrounded by a yellow medium.^11^ Isolates which showed positive growth on Mannitol Salt Agar plates were subcultured on an Oxacillin Resistant Screening Agar Base medium (Oxoid, UK) for detection of oxacillin resistance.

### Serology

PNEUMOSLIDE IgM which is an indirect immunofluorescent assay (IFA) kit (VIRCELL PNEUMOSLIDE; VIRCELL, GRANADA, Spain) for the simultaneous diagnosis in human serum of IgM antibodies of the main infectious agents of the respiratory tract was used for detection of atypical pathogens.^12^ Antibodies to *Legionella pneumophila* serogroup 1, *Mycoplasma pneumoniae, Coxiella burnetii, Chlamydophila pneumoniae*, adenovirus, respiratory syncytial virus, influenza A, influenza B and parainfluenza serotypes 1, 2 and 3 are used in this kit.^12^ However, because the aim of this study was to address the bacterial profile of LRTIs; only results for bacterial pathogens were considered and interpreted.

### Antimicrobial Susceptibility testing

Susceptibility testing was done by Disc diffusion method.^13^ The following antibiotics were tested: B-lactams (Ampicillin-sulbactam, Amoxicillin/clavulanic acid, Oxacillin), Cephalosporins (Ceftriaxone, Cefuroxime, Cefotaxime, Ceftazidime, Cefepime), Carbepenems (Imipenem), Macrolides (Erythromycin, Azithromycin, Clarithromycin), Aminoglycosides (Amikacin, Gentamicin), Quinolones (Ciprofloxacin, Levofloxacin, Moxifloxacin), and others (Vancomycin, Doxycycline). Zone diameter was measured and interpreted as per the Clinical and Laboratory Standards Institute (CLSI) guidelines.^13^

### Statistic alanalysis

Statistical analysis was carried out using the SPSS software (Ver.16).

## Results

### Patients with CAP

The predominant isolates in 360 patients with CAP were *S. pneumoniae* (36%), *C. pneumoniae* (18%), *M. pneumoniae* (12%) and *K. peumoniae* (10%). (Table 1) The sensitivity and resistance rates of *S. pneumoniae* and *K. peumoniae* against tested antibiotics are depicted in Table 2. A higher sensitivity was recorded for moxifloxacin, levofloxacin, macrolides, and cefepime; whereas, a higher rate of resistance was recorded for doxycycline, cephalosporins, ampicillin-sulbactam, and amoxicillin-clavulinate.

### Patients with HAP

The predominant isolates in 318 patients with HAP were, *methicillin-resistant Staphylococcus aureus*; MRSA (23%), *K. pneumoniae* (14%), *E. coli* (11%), *P. aeruginosa* (9%), *methicillin-sensitive Staphylococcus aureus*; MSSA (6%), and poly-microbial in 12%. (Table 1) No growth was HAP against tested antibiotics. Higher sensitivity rates demonstrated in 25%. Table 3 shows the sensitivity were recorded for vancomycin, amikacin, and resistance rates of common pathogens causative of moxifloxacin, levofloxacin, and cefepime. Characteristically, MRSA showed an absolute resistance (100%) for β-lactam-β-lactamase inhibitors, and high resistance rate (92%) for cephalosporins.

### Patients with AECOPD

The predominant isolates in 376 patients with AECOPD were *H. influenzae* (30%), *S. pneumoniae* (25%), *M. catarrhalis* (18%), *and K. pneumon*iae (12%). (Table 1) The sensitivity and resistance rates of common pathogens causative of AECOPD against tested antibiotics are demonstrated in Table 4. A higher sensitivity was noted for respiratory quinolones, macrolides and cefepime, while a higher rate of resistance was recorded for aminoglycosides, cephalosporins, and doxycycline.

## Discussion

The increasing antibiotic resistance problems, largely due to wide spread and irrational use of antimicrobial agents in hospitals and community is of great concern, especially in developing countries. Reliable statistics on antibiotic resistance that are mandatory to control spread of resistant pathogens are available from the developed nations. These data are generated by large surveillance studies in countries such as the USA, Europe, Australia, etc.^2^ However such data are sparse in developing countries -like Egypt- due to the lack of large-scale meta-analytic studies. Hospital antibiograms are commonly used to help guide empiric antimicrobial therapy and are an important component of detecting and monitoring trends in antimicrobial resistance.^6^ It would be ideal, through multicenter studies, to generate nationwide or more appropriately region-specific antibiograms.

This work represents a multicentre prospective study performed at three University Hospitals in Upper Egypt; aiming to identify the causative bacteria, antibiotic sensitivity and antibiotic resistance of LRTIs in Upper Egypt; considering this an important issue to be investigated in our locality. To the best of our knowledge, this is the first multicentre study addressing the problems of microbial aetiology, antimicrobial sensitivity and resistance of three patterns of LRTIs in Upper Egypt. International guidelines for CAP strongly recommend that locally adapted guidelines should be implemented to improve process of care variables and relevant clinical outcomes.^4^

For patients with CAP, our results showed similar bacterial profiles to those reported by the international,^4^ and local^10^ studies. However, our results showed higher prevalence of the so-called atypical organisms. This "local" pattern of predominance should be taken into consideration upon prescribing antimicrobials in our locality. Fortunately, this higher prevalence was closely-related to the susceptibility pattern; hence we found the highest rates for respiratory quinolones and macrolides. Over the past 3 decades, antimicrobial resistance among *S. pneumoniae* has escalated dramatically worldwide. By the early 1990s, penicillin-resistant clones of *S. pneumoniae* spread rapidly across Europe and globally. Additionally, resistance to macrolides and other antibiotic classes escalated in tandem with penicillin resistance. Recently, it was reported that 15 to 30% of *S. pneumoniae* worldwide are multidrug-resistant (MDR).^14^

Our data revealed high resistance rates for doxycycline, cephalosporins, and the β-lactam-β-lactamase inhibitors. These findings are in agreement with the increasing prevalence of resistance of *S. pneumoniae* to those antimicrobial groups, demonstrated by Egyptian,^15^ regional,^5^,^16^ and worldwide^4^,^5^ studies. Moreover, our results highlight the increasing problem of MDR *S. pneumoniae* in CAP, a problem that was extensively addressed in the literature.^14^–^16^ This, alarms us for the need for judicious use of different antimicrobial groups, particularly in our resource-limited country. Moreover, this calls for a greater focus on the identification of relevant drivers of resistance and on the implementation of effective strategies to combat the problems of resistance and multi-drug resistance.

With regards to patients with HAP, the problem of antibiotic resistance seems to be more important; hence the situation is more complicated than that in CAP. Nosocomial pneumonias result in high morbidity and mortality especially among ICU patients.^8^ In most clinical situations, there is a need to initiate empirical antimicrobial therapy before obtaining the microbial results. However, the situation is further complicated by the emergence of multiple beta lactamase producers and MDR pathogens.^16^,^17^ Obviously, there is a great need for obtaining data on prevalent strains in HAP; along with the susceptibility pattern, to help in revising antibiotic policy and guiding clinicians for the better management of patients with HAP; particularly in developing countries.

The current study revealed the predominance of MRSA, Gram-negative organisms, and *P. aeruginosa* among patients with HAP. This is clearly different form the results obtained by Goel and co-workers^17^ and even from those obtained by the "local" studies of Ahmed, et al^18^ and Agmy, et al.^19^ Although the later study addressed the problem of HAP in 75 cases of ICU patients at Assiut University Hospital, the predominant pathogens were *S. aureus* (32%), *P. aeruginosa* (30%), and *S. pneumoniae* (15%). Obviously, this "local" difference explains the changing pattern of causative pathogens over time, even at the same hospital. This confirms the importance of implementing continued local surveillance programmes.^1^ However, in the study of Agmy, et al,^19^ factors such as differences in patients' numbers and demographics, research methodologies, and being ICU patients should be taken into consideration. Our data show an alarming high prevalence of MRSA. This coincides with the recent report by Borg, *et al* who observed that the prevalence of MRSA in invasive isolates from blood cultures from nine hospitals in Egypt was 52%. 20 Interestingly, our data showed polymicrobial aetiology in 12% of cases; that was concordant to that reported by other studies.^17^,^21^ Our results revealed very high rates of resistance for β-lactam-β-lactamase inhibitors and cephalosporins. Goel and co-workers observed 100% and 96.9% resistance to ceftazidime against *A. baumannii*, *and Klebsiella* spp., respectively.^17^ This, again adds to the complex scenario of antimicrobial resistance found usually in nosocomial infections; particularly in developing countries.^17^,^18^ High resistance to β-lactam-β-lactamase inhibitors and cephalosporins at our locality might be due to the selective influence of overuse of these drugs. On the other hand, high susceptibility rates for respiratory quinolones still confirms the importance of these agents for management of HAP in our locality.^17^ Because MDR pathogens are usually difficult to treat and associated with increased mortality,^8^,^14^–^16^ it was recently^22^ recommended that empiric therapy of HAP requires the use of multidrug empirical treatment regimens.

Morbidity and mortality in COPD patients are, for the most part, related to their acute exacerbations, which occur one to three times a year on average.^3^ The most common causes of these exacerbations are infection of the tracheobronchial tree and air pollution.^23^ There has been a controversy regarding whether bacteria play a role in AECOPD, and thus, whether antibiotics play a role in disease management. Several studies have shown an association between the presence of certain bacterial species, such as *S. pneumoniae, M. catarrhalis and H. influenzae*, and AECOPD.^24^ However, these potential pathogenic microorganisms (PPMO) were also present in sputa obtained from COPD patients with stable disease. 25 Conversely, bronchoscopic studies have shown that at least 50% of COPD patients have bacteria in high concentrations in their lower airways during exacerbations.^26^

The profile of causative pathogens observed in the current study is very similar to that published in the literature.^3^ Recently, however, Huang and co-workers^27^ addressed the profile of airway bacterial communities using a culture-independent microarray, and concluded that bacterial community diversity in COPD airways is substantially greater than previously recognized. Further studies are needed to comprehensively profile the bacterial pathogens in AECOPD. Again, very high susceptibility rates for the respiratory quinolones confirms the importance of using such agents for AECOPD in our locality. It also represents an agreement with the international recommendations for antibiotics indicated for mild and moderate COPD exacerbations.^3^ Moreover, our reported resistance rates for aminoglycosides, cephalosporins, and doxycycline further encourage using respiratory quinolones for AECOPD in our locality. However, we still recommend the judicious use of respiratory quinolones to prevent emerging resistance to such agents.

At the end, our results in three patterns of lower respiratory tract infections have many similarities and differences to other studies. Continued surveillance; particularly based on local data is obviously needed to clarify the problems of antimicrobial resistance and to prevent further spread of such resistance.

To conclude, data from this study can be very useful. A master antibiogram for our region would allow tertiary care institutions to consider resistance patterns in hospitals referring patients and to select appropriate antimicrobial therapy or change drugs in non-responding patients. The concept of "presumptive antimicrobial therapy" could replace that of "empiric antimicrobial therapy"; based on common pathogens, known susceptibility patterns and host factors in any given region. Then, implementing continued local surveillance programs for antibiotic resistance is essentially important. Also further local studies should be carried out to elucidate the mechanisms of resistance of different pathogens in LRTIs. Judicious use of antimicrobials is essential to prevent the emergence of resistant and/or MDR bacteria in LRTIs.

## Conclusions

The most predominant bacteria for CAP in Upper Egypt are *S. pneumoniae* and atypical organisms, while that for HAP are MRSA and Gram negative bacteria. For acute exacerbation of COPD, *H. influnzae* and *S. pneumoniae* were the commonest responsible organisms. Respiratory quinolones, macrolides, and cefepime are the most efficient antibiotics in treatment of lower bacterial respiratory tract infections in our locality.

## Figures and Tables

**Table 1:** Bacterial profile of lower respiratory tract infections in Upper Egypt.

Common Bacterial pathogens (No/%)		

CAP (n=360)	HAP (n=318)	AECOPD (n=376)

*S. pneumoniae* (130/36%)	MRSA (73/23%)	*H. influnzae* (113/30%)
*C. pneumoniae* (65/18%)	*k. pneumoniae* (45/14%)	*S. pneumoniae* (94/25%)
*M. pneumoniae* (43/12%)	*E. Coli* (35/11%)	*M. catarrhalis* (68/18%)
*K. pneumoniae* (36/10%)	*P. aeruginosa* (29/9%)	*K. pneumoniae* (45/12%)
	MSSA (19/6%)	*C. pneumoniae* (19/5%)
	Poly-microbial (38/12%)	

CAP: Community-acquired pneumonia; HAP: Hospital-acquired pneumonia; AECOPD; Acute exacerbations of chronic obstructive pulmonary disease; MRSA: Methecillin-resistant *Staphylococcus aureus;* MSSA: Methecillin-sensitive *Staphylococcus aureus.*

**Table 2:** Antibiotic sensitivity and resistance rates (percentages) of *S. pneumoniae* and *K. pneumoniae* in 360 patients with CAP*

		*S. pneumoniae*	*K. pneumoniae*
**Moxafloxacin**	S	97.1	84.2
	I	0.9	4
	R	2	11.8

**Levofloxacin**	S	89.5	93
	I	4.3	2.7
	R	6.2	4.3

**Doxycycline**	S	39.9	84.4
	I	12.1	11.6
	R	48	40

**Cephalosporins**	S	56.3	50.5
	I	11.2	8.6
	R	31.5	41.4

**Macrolides**	S	82.4	87.8
	I	7.6	5.2
	R	10	7

**Ampicillin-Sulbactam**	S	52.7	53.5
	I	7.2	9.9
	R	40.7	36.6

**Amoxicillin-Clavulinate**	S	66.7	60
	I	12.8	12
	R	20.5	28

**Cefepime**	S	73.3	76.3
	I	6.7	5
	R	20	18.7

* Percentage of the number with respect to the total number of bacterial isolates of each pathogen.

CAP, community-acquired-pneumonia; S, sensitive; I, intermediate; R, resistant

**Table 3:** Antibiotic sensitivity and resistance rates (percentages) of common pathogens in 318 patients with HAP*

		MRSA	*K. pneumoniae*	*E. coli*	*P. aeruginosa*	MSSA
**Moxifloxacin**	S	44.7	74.6	73	69.5	58.9
	I	15	3.8	2.6	8	11
	R	40.3	21.6	24.4	22.5	30.1

**Vancomycin**	S	67.8				80.1
	I	12	ND	ND	ND	8
	R	20.2				11.9

**Cephalosporins**	S	8	33.3	27.2	15.5	16.7
	I	11.3	6	2	10	9
	R	80.7	60.7	70.8	74.5	74.3

**Ciprofloxacin**	S	33.3	78.4	80	75.3	42.7
	I	11	9.6	2.3	5	12.3
	R	55.7	12	17.7	19.7	45

**Cefepime**	S	44.3	61.7	65	59.9	56.6
	I	18	11.3	8.3	13.1	10
	R	37.7	27	26.7	27	33.4

**Ampicillin-Sulbactam**	S	0	36.6	48.4		51.6
	I	0	11.4	9.8	ND	14.4
	R	100	52	41.8		34

**Amoxicillin-Clavulinate**	S	0	41.2	52.7		72.2
	I	0	22.7	18.3	ND	15.4
	R	100	36.1	29		12.4

**Amikacin**	S	54.2	67.4	78	76.6	64
	I	17	8.7	10	3.4	12.6
	R	28.8	23.9	12	20	23.4

* Percentage of the number with respect to the total number of bacterial isolates of each pathogen.

HAP, Hospital-acquired-pneumonia; MRSA, Methecillin-resistant *Staphylococcus aureus;* MSSA, Methecillin-sensitive *Staphylococcus aureus;* S, sensitive; I, intermediate; R, resistant; ND, not done

**Table 4:** Antibiotic sensitivity and resistance rates (percentages) of common pathogens in 376 patients with AECOPD*

		*H. infleunzae*	*S. pneumoniae*	*M. catarrhalis*	*K. pneumoniae*
**Moxifloxacin**	s	96.6	98	94	92.2
	I	0.7	0.5	2.3	1.2
	R	2.7	1.5	3.7	6.6

**Levofloxacin**	s	94.2	94	91.7	92
	I	1.2	0.8	6.3	2.7
	R	4.6	5.2	2	5.3

**Cephalosporins**	s	87.2	55	80.4	6.76
	I	2.9	4.1	13.6	5
	R	9.9	40.9	6	28.3

**Macrolides**	s	81	79.7	77	82.2
	I	12.2	4.5	3	11.6
	R	6.8	15.8	20	6.2

**Cefepime**	s	75.6	79	80	72.5
	I	3	5.7	2.9	7.3
	R	21.4	15.3	17.1	20.2

**Aminoglycosides**	s	61.3	50	55.7	55
	I	11.6	3	8.8	11
	R	27.1	47	35.5	34

**Amoxicillin-Clavulinate**	s	74.5	68.3	77	71
	I	2.8	9.8	10.7	4.6
	R	22.7	21.9	12.3	24.4

**Doxycycline**	s	59	62	64.5	62
	I	8	6.2	9.5	6.7
	R	33	31.8	26	31.3

* Percentage of the number with respect to the total number of bacterial isolates of each pathogen.

AECOPD, acute exacerbation of chronic obstructive pulmonary disease; S, sensitive; I, intermediate; R, resistant

## List of Abbreviations

AECOPD: acute exacerbations of chronic obstructive pulmonary disease
BAL: bronchoalveolar lavage
CAP: community-acquired pneumonia
CFU: colony forming unit
CLSI: Clinical and Laboratory Standards Institute
COPD: chronic obstructive pulmonary disease
GOLD: The Global Initiative for Chronic Obstructive Lung Disease
HAP: hospital-acquired pneumonia
IFA: immunofluorescent assay
LRTI: lower respiratory tract infections
MDR: multidrug-resistant
MRSA: methicillin-resistant Staphylococcus aureus
MSSA: methicillin-sensitive Staphylococcus aureus
PPMO: potential pathogenic microorganisms
